# Subset analysis of the safety and efficacy of nivolumab in elderly patients with metastatic melanoma

**DOI:** 10.1186/2051-1426-3-S2-P133

**Published:** 2015-11-04

**Authors:** Morganna Freeman, Jeffrey Weber

**Affiliations:** 1 H Lee Moffitt Cancer Center, Tampa, FL, USA

## Introduction

Cancer immunotherapy has generated significant response rates and prolonged survival, particularly in metastatic melanoma, but carries the risk of immune-related adverse events (irAEs) [1]. Treatment of elderly patients with checkpoint inhibition presents a unique challenge as nearly half of all malignancies are diagnosed in patients >65 [2], and clinical indications for immunotherapy continue to increase. Side effects may be more challenging in older patients given the association of age with comorbidties and reduced functional reserve [3]; additionally “immunosenescence,” age-related impairment of adaptive and innate immunity, could impair effective checkpoint inhibition [4].

## Background

Retrospective analysis of irAEs and survival outcomes in melanoma patients < 65 compared to those >65 treated with nivolumab.

## Methods

Data pooled from 148 patients treated with nivolumab plus peptide vaccine or nivolumab alone every two weeks for at least 12 weeks. Frequency, grade, and characteristics of irAEs were analyzed among patients >65 and < 65 years of age. A 12-week landmark survival analysis was then assessed for each group.

## Results

Of 148 patients, 52 (35%) were age >65. Most common irAEs among pts < 65 included diarrhea/colitis (30.2%), rash (38.5%), and vitiligo (10.4%). These were also the most common irAEs in pts > 65 (21.2%, 40.4%, and 7.7%, respectively). No statistically significant difference in irAE incidence was seen between groups (p=0.322, 0.966, and 0.805, respectively) and there was no statistically significant OS difference between patients >65 and < 65 (p=0.115). A statistically significant OS benefit was seen in patients < 65 and >65 experiencing any grade of irAE (p=< 0.001 and p=0.033, respectively).

## Conclusions

Nivolumab may be an effective therapeutic option for patients 65 and older, as the irAE profile seen below and above this age cutoff was similar, and showed no statistically significant difference in incidence. Additionally the presence of irAEs in patients >65 was still associated with survival benefit, thus demonstrating such patients may still respond appropriately to checkpoint inhibition. These encouraging data should be validated in larger patient cohorts, with specifically targeted age ranges (i.e age 65-70, 70-75) to further investigate tolerance and response.

**Table 1:** Immune-related adverse events in melanoma patients <65 and >65 treated with nivolumab.

irAE	Incidence (%) Age <65	Incidence (%) Age≥65	p-value
Rash	38.5	40.4	0.966

Diarrhea	30.2	21.2	0.322

Vitilligo	10.4	7.7	0.805

Hypothyroidism	8.3	5.8	0.811

Muscositis	5.2	5.8	0.99

Myalgias	4.2	0	0.336

Pneumonitis	1.1	1.9	0.99

**Figure 1:**
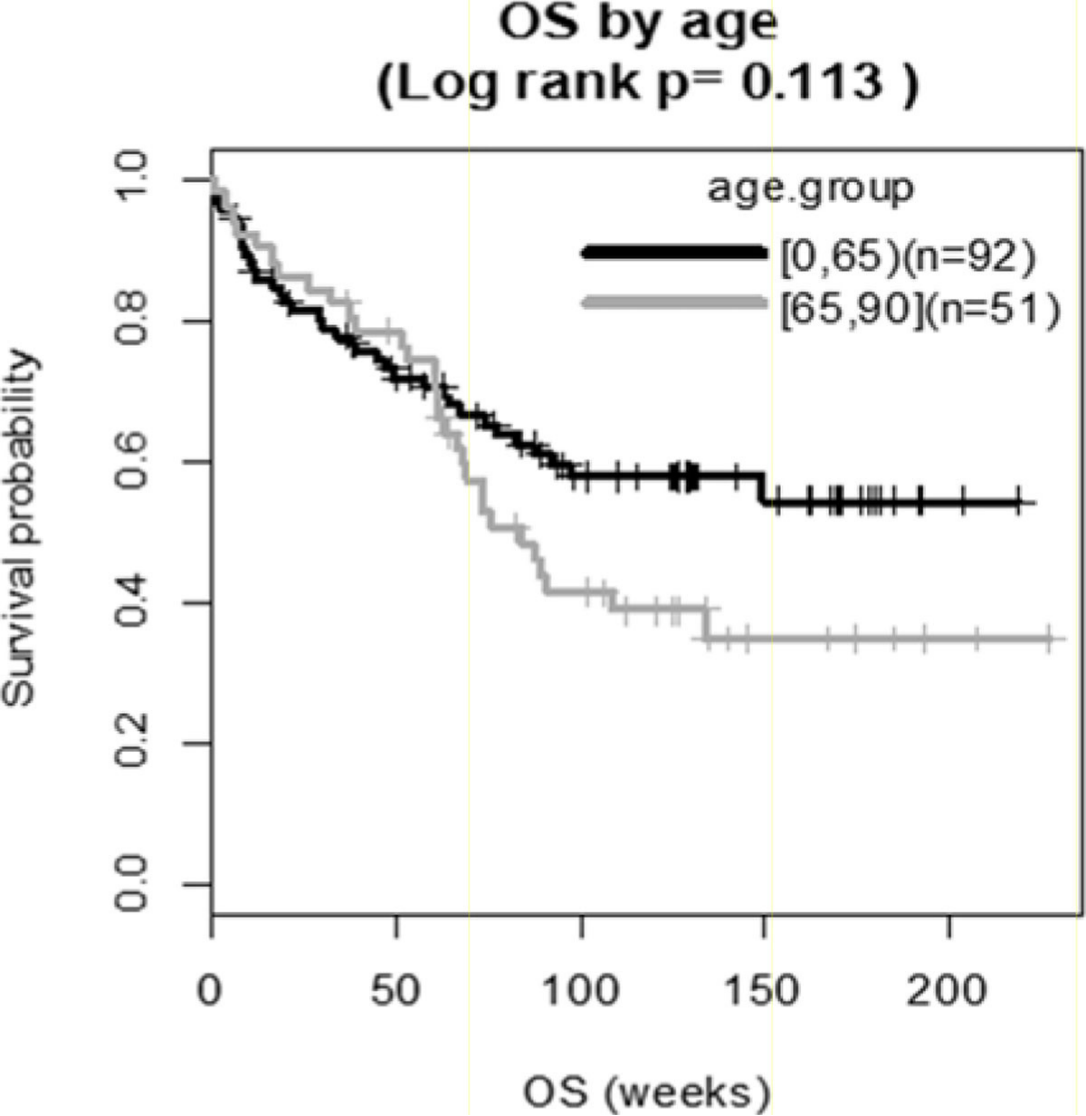
Comparison of overall survival (OS) in metastatic melanoma patients <65 and >65 treated with nivolumab.

**Figure 2:**
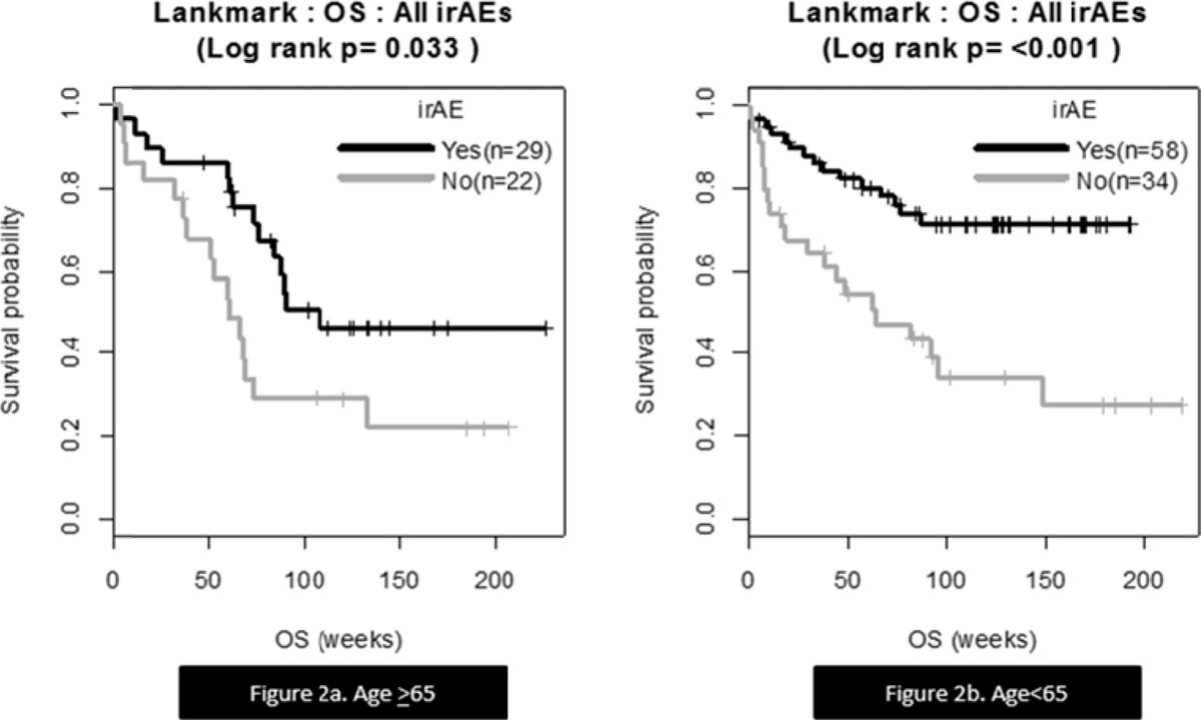
a. OS correlation to the presence or absence of irAEs in metastatic melanoma patients ≥65 treated with nivolumab. b. represents the OS curves for patients <65.

